# A systematic review of active transportation research in Africa and the psychometric properties of measurement tools for children and youth

**DOI:** 10.1186/s12966-014-0129-5

**Published:** 2014-10-18

**Authors:** Richard Larouche, Adewale L Oyeyemi, Antonio Prista, Vincent Onywera, Kingsley K Akinroye, Mark S Tremblay

**Affiliations:** Children’s Hospital of Eastern Ontario Research Institute, 401 Smyth Road, Room R242, Ottawa, ON K1H 8L1 Canada; Department of Physiotherapy, University of Maiduguri, Maiduguri, Nigeria; Physical Activity and Health Research Group, Research Center on Physical Activity and Sports, Universidade Pedagogica, Maputo, Mozambique; Department of Recreation Management and Exercise Science, Kenyatta University, Nairobi, Kenya; Nigerian Heart Foundation, Lagos, Nigeria; Department of Pediatrics, University of Ottawa, Ottawa, Canada

**Keywords:** Active travel, Motorized travel, Africa, Reliability, Validity

## Abstract

**Background:**

Previous systematic reviews indicate that active transportation (AT; the use of non-motorized travel modes such as walking, running and cycling) is an important source of daily physical activity (PA). However, no previous systematic review has examined travel behaviours among African children and youth or the psychometric properties of measurement tools used among children and youth worldwide.

**Methods:**

Studies on AT among African children and youth (aged 5–17 years) were identified through 1) the MEDLINE and Embase databases; 2) manual searches of six African journals that are not indexed in these databases; and 3) the articles included in a previous systematic review on PA among children and youth in Sub-Saharan Africa. Second, literature on the psychometric properties of measurement tools for children and youth was searched using the MEDLINE, Embase, Cochrane Central Register of Controlled Trials, PsycInfo, SportDiscus, and Health and Psychosocial Instruments databases. Study quality was assessed with a modified version of the Downs and Black checklist.

**Results:**

Twenty studies reported original data on AT among African children and youth. This evidence suggests that rates of AT to/from school are lower in urban areas and in youth attending higher SES schools. Two population-based studies reported rates of AT ranging between 19.8% and 66.6% in multiple countries. Studies conducted in Africa seldom examined non-school travel and only one reported data on the psychometric properties of their measures of travel behaviours. Nineteen studies conducted predominantly in high-income countries provided psychometric data. Child and parent reports were used in 17 studies, and these measures generally showed substantial to almost perfect test-retest reliability and convergent validity for school trips. Limited information was available regarding non-school trips. Objective measures of travel behaviours have been used much less often, and further validity and reliability assessments are warranted.

**Conclusion:**

These findings emphasize a need for more research examining travel behaviours among African children and youth, particularly for non-school travel. Further research is needed to develop valid and reliable measures of non-school travel and to examine their psychometric properties in the African context. These measures could then be used to evaluate AT promotion interventions.

## Introduction

There is ample evidence showing that the majority of children and youth are insufficiently active in order to obtain health benefits [1-4]. Of particular importance, Hallal and colleagues [3] reported that only 20% of 13–15 years olds from 105 different countries met the World Health Organization’s physical activity (PA) guidelines, which recommend that children and youth accumulate at least 60 minutes of daily moderate-to-vigorous PA [5]. Also, in a 34-country study that included 15 African countries, Guthold et al. reported that only 8% to 35% of African youth engaged in sufficient physical activity for 60 minutes a day on at least 5 days per week [6]. These large surveys emphasize that low PA levels are already occurring among youth in many African and low income countries (LIC). Because self-reported measures underestimate physical inactivity in the pediatric population [7], it is possible that the current physical inactivity pandemic in children and youth is worse than the proportion estimated in these surveys.

LIC are currently experiencing a PA transition characterized by shifts in habitual occupational PA from high-energy expenditure activities such as mining, play, hunting and gathering, forestry and farming to low-energy expenditure activities such as desk work and motorized travel [8-10]. In the African context, the effects of this transition on children and youth’s PA and health have been investigated by comparing urban areas with rural areas [9-11]. For example, there is some evidence showing that some Kenyan children living in urban areas are less fit, less active and less likely to use non-motorized travel modes such as walking and cycling (e.g. active transportation; AT) than their rural counterparts [9-11]. Because previous studies have shown that travel behaviours tend to be habitual in nature [12,13], the decision to replace AT by motorized travel may deprive children of an important source of daily PA [14].

The PA transition may have major implications for public health and the economy in LIC. Insufficient PA is associated with the development of cardiovascular disease risk factors in children and youth [15,16] and is one of the greatest causes of morbidity and mortality from non-communicable diseases, such as cardiovascular disease, cancer, and diabetes, among adults [17]. The increased prevalence of non-communicable diseases may represent an even greater challenge in LIC where the high prevalence of communicable diseases remains a major burden for healthcare systems and economic development [18-20]. In addition to these diseases, the African region has the highest road traffic fatality rate in the world, and it is estimated that 38% of these deaths occur among pedestrians [21,22].

Although the promotion of AT might be a promising strategy to simultaneously increase PA and reduce motor vehicle use and associated greenhouse gases emissions [21,23], very few studies have examined travel patterns among African children and youth. Furthermore, there remain several important gaps in the broader international AT literature. First, most studies have focused only on school trips, thereby ignoring AT to/from other destinations (i.e. parks, friends’ and relatives’ home, sport fields, etc.) which may also contribute to increase PA [24]. Second, literature reviews have noted substantial methodological heterogeneity in the measurement of school travel patterns [14,25]. Third, the psychometric properties of travel behaviour measurement tools (i.e., reliability and validity) have not been reviewed systematically; thus providing little guidance for researchers to make informed methodological decisions.

Therefore, the present systematic review aimed to provide a comprehensive summary of the AT literature among African children and youth (aged 5–17 years) and to examine the psychometric properties of travel behaviour measurement tools that have been used worldwide.

## Methods

### Search strategy

**Figure 1:**
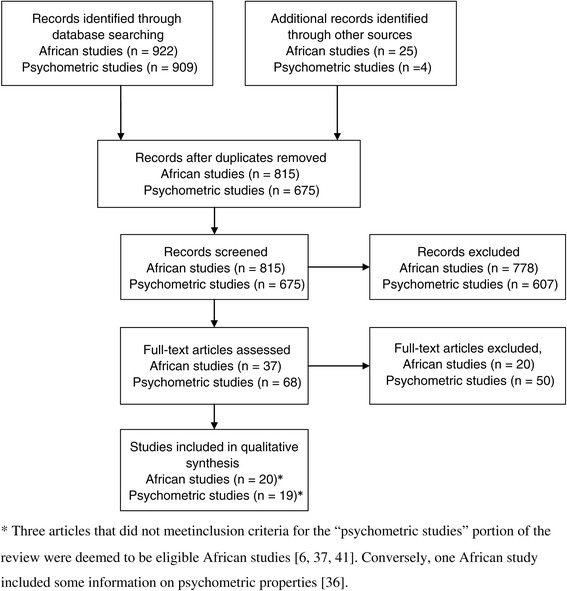
**Flow of articles in the review process.** Three articles that did not meet inclusion criteria for the “psychometric studies” portion of the review were deemed to be eligible African studies [6,27,28]. Conversely, one African study included some information on psychometric properties [29].

Two systematic search strategies were developed with the help of a professional librarian to identify potentially relevant articles published (or in press) prior to November 8, 2013. The first strategy aimed to capture African AT studies on children and youth (mean age between 5 and 17 years), and it included the articles identified through a previous systematic review on PA in sub-Saharan Africa [26]. Specifically, the search strategy of Muthuri and colleagues [26] was updated and a similar search for North Africa was run in MEDLINE and Embase. Overall, this search yielded 922 potentially relevant articles of which 790 remained after removal of duplicates. In addition, the following six African based journals which are not indexed in the databases searched were examined online: South African Family Practice; Journal of Public Health in Africa; South African Journal of Child Health; South African Journal of Clinical Nutrition; African Journal of Food, Agriculture, Nutrition and Development; and South African Journal of Sports Medicine. Two potentially relevant articles were identified this way. Furthermore, six articles were added from the review by Muthuri et al. [26], two were added from the authors’ personal libraries, and fifteen were added based on reviewers’ feedback. The second search strategy sought to identify studies reporting the psychometric properties of travel behaviour measurement tools among children and youth (hereafter referred to as “psychometric studies”) using the MEDLINE, Embase, Cochrane Central Register of Controlled Trials, PsycInfo, SportDiscus and Health and Psychosocial Instrument (HAPI) databases. This search identified 909 potentially relevant articles of which 671 remained after removal of duplicates. Two articles were added from the authors’ personal libraries, and two were added based on reviewers’ feedback. Figure 1 illustrates the flow of articles through the systematic review process.

The potentially relevant articles were imported into a Mendeley database (Elsevier, The Netherlands) and titles and abstracts were screened independently by two reviewers (RL and ALO). Full text copies of all articles that passed the initial screening were examined by two reviewers (RL and ALO) for inclusion criteria. Any disagreement was resolved with the help of a third reviewer (MST). Exceptionally, articles in Spanish (n = 1) and Portuguese (n = 1) were assessed by AP, and a French speaking researcher acted as second reviewer for French articles (n = 1). Consensus was reached for the inclusion of all included articles. The review methodology was prospectively registered in PROSPERO (Registration number: CRD42011001456).

### Inclusion and exclusion criteria

To be included in the review, African studies had to report either descriptive statistics on travel patterns among children and youth or data on the psychometric properties of travel behaviour measurement tools (i.e., reliability and validity). Non-African studies were eligible only if they reported psychometric data. The mean age of participants had to be between 5 and 17 years for studies to be included. Articles published in English, French, Portuguese, Spanish and Kiswahili were considered eligible. Only original articles were considered eligible; hence reviews, conference abstracts, commentaries and editorials were deemed ineligible.

### Data extraction

Standardized data extraction tables were created using Microsoft Excel. Information related to the study design (year, country, number of participants, age, gender, and methodology), participants’ travel patterns and the reliability and validity of the measures was extracted. For quality control purposes, data extraction was performed by two reviewers (RL and ALO) for a subsample of five articles. The percentage of agreement was 92.7% and any discrepancy was resolved by consensus. The remainder of data extraction was performed by RL, except for one article in Spanish which was assessed by AP. Reviewers were not blinded to the authors or journals when extracting data.

### Quality of evidence

The quality of the included studies was systematically evaluated using a modified version of the Downs and Black checklist [30], as shown in Table 1. In the absence of clear methodological guidance for the assessment of study quality in the context of cross-sectional studies, the Downs and Black checklist was chosen due to its suitability for the assessment of original research articles, and to the authors’ experience using this tool in other systematic reviews of observational studies [26,31,32]. Given that all included studies used cross-sectional designs, items that applied only to intervention studies (i.e. randomization process, losses to follow-up, etc.) were not retained. The modified scale included ten items pertaining to the quality of reporting (6 items), external validity (2 items) and internal validity/risk of bias (2 items). Each item was scored as “yes” (1) or “no”/“unable to determine” (0), and the sum of item scores was calculated. As shown in Table 1, three questions were considered non-applicable to qualitative studies in which no statistical tests were performed (n = 4). Hence, a 7-point quality score was used for these studies. The quality assessment was pilot tested independently by two reviewers (RL and ALO) for five studies. The initial percentage of agreement was 88.0%, and any disagreement was resolved by consensus, following which further refinement of the interpretation of the quality items were decided by the two reviewers. The remaining articles were assessed by RL.

**Table 1:** **Modified Downs and Black checklist**

***Question in the modified checklist***	***Question in the original checklist [reference*** 30 **]**	***Rating***
***Reporting***		
Q1. Objective Clearly Stated	Question 1	(Yes = 1/No = 0)
Q2. Main Outcomes Clearly Described	Question 2	(Yes = 1/No = 0)
Q3. Patient Characteristics Clearly Defined	Question 3	(Yes = 1/No = 0)
Q4. Main Findings Clearly Defined	Question 6	(Yes = 1/No = 0)
Q5. Random Variability in Estimates Provided	Question 7	(Yes = 1/No = 0)†
Q6. Actual Probability Values Reported	Question 10	(Yes = 1/No = 0)*†
***External Validity***		
Q7. Sample Targeted Representative of Population	Question 11	(Yes = 1/No = 0)
Q8. Sample Recruited Representative of Population	Question 12	(Yes = 1/No = 0)
***Internal Validity/Bias***		
Q9. Statistical Tests Used Appropriately	Question 18	(Yes = 1/No = 0)†
Q10. Primary Outcomes Valid/Reliable	Question 20	(Yes = 1/No = 0)

*This item was considered non-applicable for studies whose objectives were strictly descriptive. In addition, when actual *p* values were not reported for the outcome of interest, the score of 0 was given even though some actual *p* values were reported for other outcomes. †denotes that these questions were considered non-applicable for studies where no statistical tests were reported [33-36].

## Results

### African studies on children’s travel patterns

Titles and abstracts of 815 articles were examined of which 778 were excluded. Full text copies of the remaining 37 articles were screened in detail for inclusion criteria. Twenty articles were rejected at this stage of the review process. Of these, 17 did not include relevant data on AT, one was an earlier version of an article that is included in the present review, one was a literature review, and one was a conference abstract. Three articles from the psychometric properties search provided data on African children’s travel patterns, and were accepted for this component of the review. Thus, a total of 20 papers describing the findings of 15 different studies met inclusion criteria, and their main findings are summarized in Table 2. These studies were conducted in the following countries: Algeria [37], Kenya [9,10,38-42], Nigeria [29], Seychelles [27], South Africa [43,44], and Tanzania [45]. Two articles presented different results from the same Kenyan study [39,42]. Additionally, Guthold et al. [6] reported data from the Global School-based Student Health Survey for 15 African countries. Peltzer [28] reported more detailed analyses from this survey for four countries: Kenya, Namibia, Uganda, and Zimbabwe. Finally, 5 papers reported data from a mixed-methods research project conducted in deprived areas of Ghana, Malawi and South Africa (e.g., the Child Mobility Project) [33-36,46]. Four of these focused predominantly on qualitative data [33-36]. Sample size varied from 17 [33] to 72,845 [6] for a total number of 87,987 participants.

**Table 2:** **African studies on active transportation in children and youth**

**Lead author [reference]**	Countries*	**Sample size**	**Age or grade**	**Type of measure**	**Destinations**	**Main findings with respect to AT**
Aandstad [45]	Tanzania	156 (87B, 69G)	9-10 years	Child report	To school	90% of boys and 88% of girls walked to school. School travel time was not associated with VO_2_max as measured by a cycle ergometer test.
Bovet [27]	Seychelles	8,462 (4,239B, 4,223G)	Grades 4, 7 and 10	Child report	To/from school	In this nationally-representative sample, daily walking time was longer in children attending public schools compared to private schools. Walking to/from school was not associated with weight status.
Croteau [38]	Kenya	72 (29B, 43G)	9.8 ± 1.1 years	Child report	To school	65% of participants walked, 17% ran and 18% used IT to school. AT was associated with higher steps per day (14,924 ± 4,157 vs. 12,335 ± 2,141).
Gibson [39]	Kenya	30 (15B, 15G)	14 ± 1 years	GPS	To/from school	All participants used AT (mean distance of 7.5 ± 3.0 km/day). Boys traveled greater distances than girls (8.9 ± 2.6 vs. 6.2 ± 2.6 km/day). AT was not associated with VO_2_max as measured by indirect calorimerty.
Guthold [6]	34 countries including Botswana, Djibouti, Egypt, Ghana, Kenya, Libya, Mauritus, Morocco, Namibia, Senegal, Seychelles, Tanzania, Uganda, Zambia, and Zimbabwe	72,485 (34,674B 37,811G)	13-15 years	Child report	To/from school	Proportion of boys and girls respectively engaging in AT ≥ once per week: Botswana (53.9%; 45.1%), Djibouti (61.9%; 58.3%), Egypt (55.3%, 63.1%), Ghana (66.6%, 59.8%), Kenya (54.4%, 52.9%), Libya (62.3%, 61.1%), Mauritus (46.8%, 36.2%), Morocco (56.1%, 49.9%), Namibia (54.2%, 50.7%), Senegal (61.4%, 58.2%), Seychelles (54.8%, 49.0%), Tanzania (39.4%, 33.6%), Uganda (52.5%, 48.3%), Zambia (60.3%, 62.8%), Zimbabwe (63.8%, 54.6%)
Hampshire [46]	South Africa	959	9-17 years	Child report	To/from school and other destinations	86.6% of children walked to/from school. Of these, 31.9% reported journeys lasting > 45 min., and 8.3% had journeys > 90 min. Journeys were longer in remote rural areas. Girls were more likely than boys to travel for gathering firewood and cleaning clothes; boys played out more and went to family fields. Children living in rural areas travelled to gather water and firewood, wash clothes and work in fields more than their urban counterparts. 53.9% of participants feared dangers travelling to/from school including violence, rape and harassment (especially girls), dangerous vehicles and animals, rivers to cross and rough terrain.
Larsen [40]	Kenya	30 B	16.6 ± 0.8 years	Child report	To/from school	Village boys ran greater distances between home and school than town boys (0.9 vs. 0.3 km/day), but no differences were found for walking.
Lennox [43]	South Africa	318 (137 B, 181 G)	Grade 8	Child report	To school	In a low SES school, 96.4% of youth walked to school, of whom 63.9% reported distances >3 km. In an higher SES school, 92.4% walked to school, of whom 98.5% reported distances <2 km. Higher reported distance between home and school was associated with greater PA (particularly in boys).
Muthuri [41]	Kenya	563 (262 B, 301 G)	9-11 years	Child report	To/from school	45.7% of participants engaged in AT. They were less likely to be overweight/obese (14.7 vs. 25.8%) and more likely to meet PA guidelines compared to those that used IT (22.4 vs. 5.5%). These associations were NS in fully-adjusted models.
Ojiambo [9]	Kenya	200 (98B, 102G)	13.0 ± 1.0 years	Child report	To school	All rural youth engaged in AT (40% walking, 60% running), whereas 50% of urban youth where driven by car, 41% walked and 9% ran. All urban youth had school journey times <30 minutes while 52% of rural youth had journeys ≥30 minutes.
Ojiambo [42]	Kenya	30 (15B, 15G)	14 ± 1 years	GPS	To/from school	All participants used AT (mean distance of 7.5 ± 3.0 km/day). AT distance was not associated with PA (as measured by doubly-labeled water) and BMI z-score.
Onywera [10]	Kenya	169 (85B, 84G)	9-12 years	Parent report	To/from school	87% of rural children engaged in AT (58% walking and 29% running) vs. 42% of urban children (41% walking and 1% running). 99% of rural parents engaged in AT as a child vs. 89% of urban parents.
Oyeyemi [29]	Nigeria	1,006 (499 B, 507 G)	15.6 ± 1.7 years	Child report	To school	Participants reported engaging in AT 61.9 min/week (boys: 72.5 min vs. girls: 51.4 min; p = 0.002). Perceived access to destinations (e.g., schools, shops to buy things, and bus stops) was associated with greater engagement in AT in boys, but not in girls. Other built environment constructs were not associated with AT.
Peltzer [28]	Kenya, Namibia, Uganda, Zimbabwe	12,740 (6,039B, 6,701G)	13-15 years	Child report	To/from school	Proportion of youth engaging in AT ≥5 days/week in Kenya, Namibia, Uganda, Zimbabwe were respectively 24.9%, 19.8%, 27.9% and 31.1%. Compared to Kenya, Namibian youth were less likely to engage in AT (OR = 0.74), but Ugandan (OR = 1.16) and Zimbabwean (OR = 1.36) youth were more likely. Females were less likely than males, but effect size was trivial (OR = 0.98; R^2^ = 0.00).
Porter [33]	Ghana, Malawi and South Africa	17 (6B, 11G)	11-22 years	Ethnographic interviews	From school	All children walked from home to school, covering a distance of about 5 km. Girls tended to be more afraid about encounters with strangers. They spent more time doing household tasks before school, which often led to late arrival, punishment, truancy and school dropout. Younger children found the journey more physically difficult and dangerous.
Porter [34]	Ghana, Malawi and South Africa	2,967 + 50-80 interviews per site (n = 24; 8 per country)	9-18 years	Child report, interviews, life histories, focus groups, ethnographic diaries, accompanied walks	To/from school and other destinations	Proportion of boys and girls respectively walking to/from school: Ghana (97.4%, 98.6%), Malawi (99.1%, 99.3%), South Africa (86.4%, 86.3%). Proportion of boys and girls respectively carrying water: Ghana (82%, 71%), Malawi (23%, 55%), South Africa (26%, 38%). While children were rarely accompanied by adults, they typically traveled in same-gender groups. Girls’ mobility beyond trips to/from school and household chores was restricted by their parents, especially after they reached puberty; boys were granted more independent mobility.
Porter [35]	Ghana, Malawi and South Africa	2,967 + interviews (*N* not specified)	9-18 years	Child report, interviews, life histories, focus groups, ethnographic diaries, accompanied walks	To/from school	Same rates of walking to school as in Porter et al. (2010b) [31]. Proportion of boys and girls living in remote rural areas who respectively felt safe on their trip to/from school: Ghana (44%, 31%), Malawi (45%, 42%), and South Africa (21%, 24%). Participants’ concerns about traffic, attacks from people, rape, rough terrain and rivers to cross differed between gender and countries.
Porter [36]	Ghana	1,005 + 150 interviews	9-18 years	Child report, interviews, life histories, focus groups, ethnographic diaries, accompanied walks	To/from school	98.6% of girls and 97.4% of boys walked to/from school, traveling distances up to 10 km. Teachers reported that ~70% of children miss school in the rains because they most cross an unbridged river. Large proportions of boys and girls were afraid of dangerous animals and attacks from people. Household work burden (which often involve long journeys on foot) often leads to late school arrival and consequent punishment (including corporal punishment), particularly for girls.
Taleb [37]	Algeria	912 (462B, 450G)	9.6 ± 2.0 years	Child report	To/from school	93% of normal weight children and 90% of overweight children walked to school (NS). Distance and school travel time did not differ by weight status.
Walker [44]	South Africa	240 (120B, 120G)	10-12 years	Child report	To/from school	All participants walked to/from school. Height and weight did not differ between children living closer or further from school. Boys who traveled longer distances had higher HDL cholesterol (1.83 vs. 1.71 mmol/L), but no such association was observed in girls. Distance was not associated with total cholesterol and triglycerides.

**Note**: All included studies used cross-sectional designs. *Only data from African countries are considered eligible for this section of the review, so non-African countries are not listed. B = boy; G = girl; AT = active transportation; IT = inactive transportation; GPS = global positioning system; NS = non-significant; PA = physical activity.

For quantitative studies (n = 16), quality score ranged from 4 to 8 with a mean of 6.1 out of 10 (Table 3), indicating modest quality. The four qualitative studies received an average score of 3.3 out of 7.0. However, only one of the 20 studies reported any data on the reliability and validity of their travel behaviour measurement tools. Specifically, Oyeyemi et al. [29] used AT questions that exhibited moderate test-retest reliability (ICC =0.45) in a subsample of Nigerian adolescents (Adewale Oyeyemi, personal communication). Oyeyemi et al. [29] also examined adolescents’ environmental perceptions using an adapted version of the Physical Activity Neighborhood Environment Scale that has shown good reliability and construct validity in a Nigerian sample [47]. In addition, only three included papers were based on representative samples [6,27,28]. Therefore, most studies received scores of 0 for the two external validity items. Finally, in many cases, it was unclear how AT was assessed raising concerns about the reproducibility of the studies.

**Table 3:** **Study quality assessment**

**Author (year)**	**Q1**	**Q2**	**Q3**	**Q4**	**Q5**	**Q6**	**Q7**	**Q8**	**Q9**	**Q10**	**Total score**
**1. a) Quantitative African studies (mean)**	**1**	**1**	**0.75**	**0.81**	**0.94**	**0.40**	**0.25**	**0.13**	**0.81**	**0**	**6.06/10**
Aandstad [45]	1	1	0	1	1	0	0	0	1	0	5/10
Bovet [27]	1	1	1	1	1	0	1	1	1	0	8/10
Croteau [38]	1	1	1	1	1	1	0	0	0	0	6/10
Gibson [39]	1	1	1	1	1	0	0	0	1	0	6/10
Guthold [6]	1	1	1	1	1	N/A	1	1	1	0	8/9
Hampshire [46]	1	1	1	1	0	0	0	0	1	0	5/10
Larsen [40]	1	1	1	0	1	0	0	0	1	0	5/10
Lennox [43]	1	1	1	0	1	0	0	0	0	0	4/10
Muthuri [41]	1	1	1	1	1	1	0	0	1	0	7/10
Ojiambo [9]	1	1	1	1	1	1	0	0	1	0	7/10
Ojiambo [42]	1	1	0	1	1	0	0	0	1	0	5/10
Onywera [10]	1	1	1	1	1	1	0	0	1	0	7/10
Oyeyemi [29]	1	1	1	1	1	1	1	0	1	0†	8/10
Peltzer [28]	1	1	0	1	1	0	1	0	1	0	6/10
Taleb [37]	1	1	1	0	1	1	0	0	0	0	5/10
Walker [44]	1	1	0	1	1	0	0	0	1	0	5/10
**1. b) Qualitative African studies**	1	1	0.25	1	N/A	N/A	0	0	N/A	0	3.25/7
Porter [33]	1	1	1	1	N/A	N/A	0	0	0	N/A	4/7
Porter [34]	1	1	0	1	N/A	N/A	0	0	0	N/A	3/7
Porter [35]	1	1	0	1	N/A	N/A	0	0	0	N/A	3/7
Porter [36]	1	1	0	1	N/A	N/A	0	0	0	N/A	3/7
**2. Psychometric studies (mean)**	**1**	**0.95**	**0.74**	**1**	**0.53**	**0.26**	**0**	**0**	**1**	**0.89**	**6.37/10**
Bere [48]	1	1	1	1	0	1	0	0	1	1	7/10
Brug [49]	1	1	1	1	1	0	0	0	1	1	7/10
de Wit [50]	1	1	0	1	1	0	0	0	1	1	6/10
Ducheyne [51]	1	0	1	1	0	0	0	0	1	1	5/10
Evenson [52]	1	1	1	1	1	0	0	0	1	1	7/10
Evenson [53]	1	1	1	1	1	0	0	0	1	1	7/10
Heelan [54]	1	1	1	1	0	0	0	0	1	1	6/10
Hermoso [55]	1	1	0	1	0	0	0	0	1	1	5/10
Kelly [56]	1	1	0	1	1	0	0	0	1	1	6/10
Larouche [57]	1	1	0	1	1	1	0	0	1	1	7/10
McDonald [58]	1	1	1	1	1	0	0	0	1	1	7/10
Mendoza [59]	1	1	0	1	0	1	0	0	1	1	6/10
Murtagh [13]	1	1	1	1	0	0	0	0	1	1	6/10
Oyeyemi [29]	1	1	1	1	0	0	0	0	1	0†	5/10
Philippaerts [60]	1	1	1	1	1	0	0	0	1	0	6/10
Rodriguez [61]	1	1	1	1	1	0	0	0	1	1	7/10
Rosenberg [62]	1	1	1	1	0	1	0	0	1	1	7/10
Singh [63]	1	1	1	1	0	0	0	0	1	1	6/10
Suminski [64]	1	1	1	1	1	1	0	0	1	1	8/10

Note: study quality was assessed with a modified version of the Downs and Black [30] checklist, as detailed in Table 1. The Q1 to Q10 columns refer to the questions listed in Table 1. †The quality assessment was based on the information provided in the article, rather than the test-retest reliability coefficient obtained by personal communication.

Seventeen studies assessed travel patterns with questionnaires. Questionnaires were completed by the participants in sixteen studies [6,9,27-29,34-38,40,41,43-46] and by their parents in one study [10]. Four papers from the Child Mobility Project complemented their questionnaire findings with rich qualitative data collected using a variety of methods (individual interviews, focus groups, life histories, ethnographic diaries and accompanied walks) [34-36,46]. In one study, ethnographic interviews were conducted with children walking home from school [33]. Fourteen studies reported data on school travel mode [6,9,10,28,33-38,37,41,43,45,46], eight reported on duration [27,29,33,34,37,38,45,46] and seven reported on distance traveled [33,35-38,40,42]. The proportion of children engaging in AT to/from school varied markedly across studies from 19.8% (e.g., the percentage of children reporting walking or biking to school ≥5 days per week in Namibia [27]) to 100% [9,42] (only for participants living in rural areas of Kenya and South-Africa).

Two papers from the Child Mobility Project investigated non-school journeys [34,46]. Hampshire et al. [46] reported the percentage of South African children reporting journeys for various purposes including fetching water and firewood, going to wash clothes, buying/selling items at the market, going to faith places, working in fields, and paying social visits. Youth living in rural settlements were more likely to travel to fetch water and firewood, to work in fields, and to wash clothes, while their urban counterparts traveled more for social visits. Porter et al. [34] reported data on the proportion of boys and girls who traveled to carry water in these 3 countries (Table 2), and provided a detailed account of youth’s mobility. Very few children were accompanied by adults on their trips. Instead, children typically traveled in same-gender groups as parents believed that this strategy reduced the likelihood of attacks from people, and sex-related temptations. After the onset of puberty, girls’ mobility was severely restricted, particularly for non-utilitarian trips and after dark, as parents were concerned that they would get pregnant [34].

Three studies examined differences in school travel mode between urban and rural areas, reporting higher rates of AT in the latter [9,10], or greater running distance in rural youth [40]. Moreover, Onywera et al. [10] asked parents to report how they traveled to/from school as a child and how their children travel to/from school. 99% of rural Kenya and 89% of urban Kenya parents engaged in AT as a child, while corresponding figures for their own children were 87% and 42%. In addition, two studies reported that children attending private school (used as a proxy for individual socioeconomic status) had shorter walks to/from school [27,43]. Muthuri et al. [41] also noted that children attending private schools were much less likely to engage in AT than those attending public schools.

Oyeyemi et al. 29] examined the association between built environment characteristics and travel behaviour in adolescents living in Maiduguri, Nigeria. They noted that boys who perceived greater access to neighborhood destinations spent more time engaging in AT, but this relationship was not significant for girls. No other built environment characteristics were associated with AT. The Child Mobility Project provides a detailed overview of the various challenges faced by children on their school journeys. 53.9% of South African participants expressed concerns about dangers on their route to/from school including attacks from people, dangerous animals, dangerous vehicles, unbridged rivers to cross, rough terrain, rape and harassment [46]. Such concerns were also common among participants from Ghana and Malawi [34-36]. South African girls were much more likely to be afraid of rape and harassment than boys, and many interviewed girls reported experiences of unwanted love proposals while travelling to/from school [46].

Potential benefits of AT were examined in seven of the studies that used self-reported measures [27,37,38,41-43,45]. First, Croteau et al. [38] noted that children who walked or ran to school accumulated 2,589 additional pedometer steps per day. Lennox et al. [43] found that greater distance between home and school was associated with higher self-reported PA. However, three studies found no relationship between AT and weight status [27,37,44]. Aandstad et al. [45] found no association between school travel time and cardiovascular fitness as measured by a cycle ergometer test. In contrast, Walker and colleagues [44] reported that boys who traveled longer distances had significantly greater high density lipoprotein (HDL) cholesterol levels, but this association was not observed in girls, nor was distance traveled associated with total cholesterol and triglycerides. Finally, Muthuri et al. [41] noted that active travelers were less likely to be overweight or obese (14.7% vs. 25.8%) and more likely to meet PA guidelines (22.4% vs. 5.5%) than inactive travelers. These differences were no longer significant after adjusting for the type of school (e.g., public vs. private).

Lastly, two articles reported results from the same study in which global positioning systems (GPS) were used to measure the distance between home and school among Kenyan youth who all walked or ran to/from school [39,42]. These children traveled long distances to/from school (7.5 ± 3.0 km/day), with significantly longer distances in boys compared to girls (8.9 ± 2.8 vs. 6.2 ± 2.6 km/day). However, AT distance was not significantly associated with cardiovascular fitness (e.g., VO_2_max), BMI z-score, or doubly labeled water-measured energy expenditure and PA level.

### Studies reporting psychometric properties of travel behaviour measures

Titles and abstracts of 675 articles were examined of which 607 were excluded. Full text copies of the remaining 68 articles were screened in detail for inclusion criteria. Fifty articles were rejected for the following reasons: no original data on the reliability or validity of AT questions (40 papers), ineligible exposure (e.g. no measure of AT per se; 7 papers), and ineligible age range (7 papers). Some papers were rejected for more than one of these reasons. One of the African studies included a test-retest reliability assessment [29]. Therefore, 19 studies presented original research findings related to the psychometric properties of active transportation measurement tools among children and youth and were included in this component of the review (Table 4). Eight studies were conducted in the United States [52-54,58,59,61,62,64], two in Belgium [51,60], and one in each of the following countries: Canada [57], New Zealand [50], Nigeria [29], Norway [48], Scotland [13], Spain [55] and United Kingdom [56]. Two studies were simultaneously conducted in Belgium, Greece, Hungary, the Netherlands, Norway, Slovenia and Spain [49,63]. Study quality ratings ranged from 5 to 8, with a mean of 6.4. None of the psychometric studies were done with representative samples, so they were given scores of zero for the two external validity items. In addition, several studies did not report measures of random variability (i.e., confidence intervals) and *p* values for the reliability and validity estimates.

**Table 4:** **Overview of studies reporting on the psychometric properties of active transportation measurement tools**

**Lead author [reference]**	**Countries**	Sample size*	**Age or grade**	**Type of measure**	**Destinations**	**Psychometric data**
Bere [48]	Norway	106 (39B, 67G)	11-12 years	Child report	To/from school	Test and retest conducted 14 days apart. Children reported the number of trips to/from school they do by walking, cycling, car and bus in a usual week. Spearman correlations were 0.85-0.92 for the number of school trips made by each mode. Kappa of 0.93 for the determination of main school travel mode based on reported frequency.
Brug [49]	Belgium, Greece, Hungary, the Netherlands, Norway, Slovenia, Spain	720 (for test-retest)	11.6 ± 0.7 years	Child report	To/from school	Test and retest conducted one week apart. ICC was reported for the mode of transport on previous day (0.79), and the number of minutes cycled (0.81) or walked (0.70) to school
de Wit [50]	New Zealand	118 + parents	7.1 ± 1.6 years	Child and parent report	To school	Test-retest reliability (3–4 hours apart): percent agreement (97%); kappa for child reports (0.96; 95% CI = 0.92-1.00). Convergent validity between child and parent reports: percent agreement (93%); kappa (0.91; 95% CI = 0.85-0.98)
Ducheyne [51]	Belgium	69 (41B, 27G) + parents	10.5 ± 1.1 years	Child and parent report	Cycling to/from school and other destinations	Test and retest conducted one week apart. ICC = 0.94 for child reported school travel time and distance. Corresponding ICCs for parent reports were 0.96 and 0.97. ICCs for the number of non-school cycling trips on weekdays and weekends days were 0.44 and 0.64. Concurrent validity: Pearson correlation between child reported distance and shortest car path distance was 0.45
Evenson [52]	United States	480 (all girls)	Grades 6 and 8	Child report	To school	Test and retest conducted a median of 12 days apart. ICC for the number of days engaging in AT in the past week = 0.60 (95% CI = 0.52-0.67).
Evenson [53]	United States	†	8-11 years	Child and parent report	To/from school	Test-retest reliability one day apart. Kappa = 0.79-1.00 for items on travel mode to and from school, accompaniment, and number of walk or bike trips during the week. Convergent validity between child and parent reports: kappa = 0.80 (0.71-0.89) for school travel mode and ICC = 0.55 (0.24-0.76) for number of AT trips during the week
Heelan [54]	United States	320 (141B, 179G) + parents	10.2 ± 0.7 years	Child and parent report	To/from school	Test and retest conducted 2 days apart. Percentage agreement between child reports = 97%. Convergent validity of child and parent reports = 97.5%. Correlation of distance between children’s home and school estimated by Expedia.com and measured distance (Rolatape measuring wheel) = 0.91.
Hermoso [55]	Spain	291 (139B, 152G)	9-12 years	Child report	To/from school	Test and retest conducted 14 days apart. The study examined 3 different seasons. Kappa values ranged between 0.81 and 0.87 and percent agreement form 91% to 93% with no difference between genders and seasons.
Kelly [56]	United Kingdom	17 (6B, 11G)	13-15 years	Wearable camera + child report	All active trips	On average, self-report journeys were 10 seconds longer than objectively measured (95% CI = −33 to 53), but the Bland-Altman limits of agreement were large (±501 seconds). Inter-rater reliability: kappa = 1.00 for coding travel mode and 0.99 for coding trip duration based on the camera. Convergent validity: correlation between self-reported and objectively measured journey times within-subject and between-subject = 0.89 and 0.92.
Larouche [57]	Canada	22 (9B, 13G)	10-14 years	Child report	To/from school	The volume of AT to/from school calculated by multiplying the number of reported active trips per week by the home-school distance estimated with Google Maps. Test and retest conducted 1 week apart. ICC = 0.87 for the volume of AT to/from school; Kappa = 0.77 for the classification of individuals as active vs. inactive travelers based on reported trip frequency.
McDonald [58]	United States	542 + parents	Kinder-garten to Grade 5	Child and parent report	To/from school	Test and retest for child and parent surveys conducted 1 day and 1 week apart respectively. Test-retest reliability: kappa for child-reported travel mode to and from school was 0.86 and 0.85 respectively. Kappa for the reliability of parent survey questions on travel mode, time and distance varied between 0.62 and 0.97. Convergent validity between child- and parent-reported travel mode to/from school: kappa ≥0.77.
Mendoza [59]	United States	97 (42B, 55G)	Grade 4	Child and parent report	To/from school	Test and retest conducted 4 hours apart. Test-retest reliability: kappa = 0.97. Convergent validity between child- and parent-reported travel mode was moderate (kappa = 0.52), but when combining the car and carpool survey options, it was much higher (kappa = 0.87)
Murtagh [13]	Scotland	126 (74B, 52G)	8-9 years	Step counts of the school trip	To/from school	Actigraph accelerometer step counts for 4 trips to and from school were averaged (mean = 2,262 steps); the internal consistency (Cronbach α) was 0.87. This measure was significantly predicted by children’s intention to engage in AT and habit strength (assessed in preceding week).
Oyeyemi [29]	Nigeria	56 (25B, 31G)	12-18 years	Child report	To school	Test and retest conducted over 2 consecutive weeks. ICC for the number of min/week of AT to school reported by youth was 0.45. [Adewale Oyeyemi, personal communication].
Philippaerts [60]	Belgium	33 (10B, 23G)	14.4 ± 1.4 years	Child report	To/from school and other destinations	Test and retest conducted 8 days apart. AT time was calculated as the sum of the reported time spent walking and cycling for transport. ICC for weekly school travel time, non-school travel and total travel were respectively 0.84, 0.72 and 0.72. Concurrent validity: reported AT time showed non-significant correlations with CSA accelerometer outputs.
Rodriguez [61]	United States	51 (all girls)	Grades 10 and 11	GPS/accelerometer and child report	All walking trips	7 algorithms were used to identify walking trips from the combination of GPS and ActiGraph accelerometer. Concurrent validity: agreement between self-reported and GPS/accelerometer identified trips ranged from 0.33-0.48 at the person-day level (e.g., for the number of trips/day) and from 0.41-0.64 at the person level (e.g., mean number of trips/day). Agreement ranged between 86.4 and 100% for the location of trips that were both self-reported and identified.
Rosenberg [62]	United States	287 + parents	Children (5–11) and youth (12–18)	Child and parent report	Walking to/from school, parks and shops	When ≥1 walking trip to a given destination was reported, participants were classified as “walkers” for this destination. Reliability was not reported for the walking questions. Predictive validity: several relationships between walking and Neighborhood Environment Walkability Scale for Youth subscales were noted for all destinations based on both parent and youth reports (Rosenberg et al., [62]; Tables three–five).
Singh [63]	Belgium, Greece, Hungary, the Netherlands, Norway, Slovenia, Spain	826	10-12 years	Child report	To/from school	Test and retest conducted 1 week apart (n = 730). ICC for 7 items on school travel mode and duration range from 0.70 to 0.94. Construct validity was examined by conducting interviews with a separate sample (n = 96). Following the interview, research staff completed the questionnaire based on the recorded and transcribed interviews. The child and staff questionnaires were compared (ICC = 0.59 to 0.84).
Suminski [64]	United States	N/A‡	Kinder-garten to Grade 5	Direct observa-tion	To/from school	Study staff observed 3 school entry points in 2 different schools for 3 days/week over 8 weeks during the 30 minutes before and after school. One day of measurement provided a reliable estimate of the number of children walking (r = 0.83; 95% CI = 0.61-0.97), while two days provided a better estimate (r = 0.97; 95% CI = 0.92-1.00). Inter-rater agreement for the number of children walking to and from school was 97% and 97.5% respectively. Bike trips were too infrequent to estimate reliability.

**Note**: All included studies used cross-sectional designs for the assessment of reliability and validity. *The term “+ parents” is mentioned in the sample size column when parents of participants acted as “criterion” for the assessment of convergent validity. †In this study, separate samples were used for test-retest reliability (n = 54), and convergent validity (n = 28). ‡This study used direct observation, and there were no participants. B = boy; G = girl; AT = active transportation; IT = inactive transportation; GPS = global positioning system; PA = physical activity; ICC = intra-class correlation coefficient.

Questionnaires were the most common tool used to assess travel patterns (17 studies). These instruments were completed by children only in 10 studies [29,48,49,52,55-57,60,61,63] or by both children and parents in 7 studies [50,51,53,54,58,59,62]. Of note, Kelly et al. [56] used a combination of child reports and wearable camera, and Rodriguez et al. [61] combined child reports with GPS and accelerometers. Additionally, Murtagh et al. [13] estimated the step counts associated with school trips based on the ActiGraph accelerometer, and Suminski et al. [64] used direct observation at two different schools. Thirteen studies focused only on the school trip [13,48-50,52-55,57-59,63,64]. Data on participants’ travel mode(s) were reported in 16 studies [48-59,61-64]. Seven studies reported data on trip duration [49,51,56,58,60,61,63]. Questions on school travel distance were asked in two studies [51,58], while distance was estimated using internet programs in two studies [54,57]. Finally, participants were asked to report the amount of time that they spent engaging in AT in 3 studies [29,49,60].

Fifteen studies assessed the test-retest reliability of AT measures [29,48-55,57-60,63,64], that is the degree of similarity between repeated measures taken among the same individuals under the same conditions [65]. Almost all of these studies reported substantial (0.6-0.8) to almost perfect (0.8-1.0) reliability for survey items pertaining to school trips based on the cut-points of Landis and Koch [66]. Oyeyemi et al. [29] noted moderate test-retest reliability (ICC = 0.45) for the number of minutes per week of AT to school reported by Nigerian youth. The reliability of items targeting a broader range of destinations was assessed in only two studies. Ducheyne et al. [51] reported intra-class correlation coefficients (ICCs) of 0.44 and 0.64 for the number of non-school cycling trips on weekdays and weekend days respectively. Philippaerts et al. [60] reported ICCs of 0.72 for the child-reported number of hours per week of overall and leisure time active transportation. The interval between test and retest ranged from 3–4 hours to 14 days, with shorter intervals generally associated with greater reliability coefficients (Table 4). One study examined the internal consistency of a measure of the number of steps taken during 8 journeys to/from school, and reported a Cronbach alpha of 0.87 [13]. This indicates that the amount of steps taken during each journey was strongly correlated. Kelly et al. asked 17 participants to wear a Microsoft SenseCam wearable camera for their school journeys over one week, then the researchers coded participants’ travel mode and trip duration [56]. There was almost perfect inter-rater reliability for both travel mode and duration, indicating that different researchers were consistent in their coding. Similarly, using a direct observation method, Suminski et al. [64] reported almost perfect inter-rater reliability for coding children’s school travel mode.

Multiple measures of validity were used. First, convergent validity refers to the degree to which two measures of constructs that should theoretically be related, (i.e. child- and parent-reported school travel) are in fact related. Convergent validity was examined in five studies [50,53,54,58,59], and most showed very high correlations. However, an ICC of 0.55 was noted by Evenson et al. [53] for the number of active trips to/from school during the week. Mendoza et al. [59] reported a kappa of 0.52 for reported school travel modes, but when the response options “car” and “carpool” were combined, the kappa was 0.87. Second, four studies reported on the concurrent validity of different measures [51,56,60,61], that is the extent to which two different measures of the same construct are correlated. Ducheyne et al. [51] reported that the Pearson correlation between child-reported distance and the shortest car path distance measured by the Mappy program was 0.45. Kelly et al. [56] noted that although reported and observed trip duration differed on average by only 10 seconds, there were very wide Bland-Altman 95% limits of agreement. Philippaerts et al. [60] found no significant correlations between child-reported AT time and CSA accelerometer output while Rodriguez et al. [61] noted moderate agreement between the number of walking trips reported by adolescents and the number of trips identified through a combination of GPS and accelerometers. Third, one study indicated substantial construct validity of school travel questions [63], indicating that the questions measure what they claim to be measuring [65]. Finally, two studies examined predictive validity [13,62], that is the degree to which a measure predicts another variable that it should theoretically be able to predict [65]. Rosenberg et al. [62] noted several associations between walking to/from school, parks and shops and perceived built environment constructs (measured with the Neighborhood Environment Walkability Scale for Youth). Murtagh and colleagues [13] reported that children’s intention to engage in AT to/from school and their habit strength predicted the average number of accelerometer-measured steps that they took during school trips in the following week.

## Discussion

The present systematic review provides a comprehensive overview of the AT literature on children and youth in Africa as well as the psychometric properties of travel behaviour measurement tools that have been used worldwide among children and youth. 20 studies as indicated above examined the travel patterns of African school children. Only one of them examined the psychometric properties of its instruments and very few studies reported data on trips to/from destinations other than school. Nevertheless, the included studies provide preliminary evidence suggesting that rates of AT to/from school are lower in urban areas and in higher SES schools. Nineteen studies conducted predominantly in high income countries presented data on the psychometric properties of travel behaviour measurement tools. Child and parent reports were used most frequently and these measures generally had substantial to almost perfect test-retest reliability relative to school travel. Convergent validity between child and parent reports was generally substantial, but findings for concurrent validity between different measures were conflicting. Finally, very few studies have used objective measures of travel behaviours and considered destinations other than school.

### African studies on children’s travel patterns

The preliminary findings of the African studies are generally supportive of the PA transition concept whereby high-energy expenditure activities are gradually being replaced by low-energy expenditure activities, including, among other things, motorized travel [8,26,31]. Of particular interest, using nationally-representative data from 15 African countries, Guthold and colleagues [6] noted that between 33.6% and 66.6% of youth reported walking or cycling to/from school at least once per week. In comparison, rates of cycling to/from school ≥60% are often reported among youth in Northern European countries such as Belgium [67] and Denmark [68-71]. It appears that the rates of cycling to/from school reported in the European countries of Belgium and Denmark are higher (even when omitting walking) than the combined rates of walking and cycling to/from school reported in many African countries. Using a different classification than Guthold et al. [6], Peltzer [28] reported that between 19.8% and 31.1% of 13–15 year olds from Kenya, Uganda, Zambia and Zimbabwe reported engaging in AT to/from school on at least 5 days/week. Interestingly, a similar proportion of Canadian parents interviewed as part of the National Longitudinal Survey of Children and Youth declared that their 13–15 year olds usually engaged in AT [72]. Finally, Hampshire et al. [46] observed in South-Africa that, compared to youth living in rural areas, fewer youth living in urban areas traveled to fetch water and firewood, to work in fields, and to wash clothes. Collectively, these findings underscore that more efforts are needed to promote AT among African children and youth, especially in urban and higher SES areas.

Interestingly, the observation that AT to/from school is less common in urban Kenya compared to rural Kenya [9,10] is in contrast with the North American literature generally reporting much higher rates of AT to/from school in urban areas [72,73]. This difference may be explained by the increasing availability of motorized vehicles in urban areas as posited by the PA transition model [8]. Driving a vehicle may also be perceived as a sign of prestige and prosperity leading individuals to use cars even for short trips, despite the notoriously slow speed of motorized travel in cities such as Nairobi [74]. Comparatively, access to motorized vehicles may be very limited in rural areas, so these individuals may have little option other than to travel by non-motorized means. Motorized travel may also provide a way to avoid dangers on the route to/from school. For instance, Hampshire et al. [46] and Porter et al. [33-36] described the concerns related to violence, rape, robbery, traffic, wild animals, and the risk of drowning while fording rivers on the route to/from school in the rainy season. They also emphasized the burden of household chores, particularly for girls living in rural areas who often have to walk long distances to gather water and firewood before going to school, which can make the trip to school daunting and lead to truancy and dropout.

Apart from the differences in AT between rural and urban youth, the included studies provide little detail on the correlates of AT in Africa. A better understanding of such correlates is needed to inform the development of effective interventions to promote AT (and/or prevent the decrease in AT associated with the PA transition). The international literature highlights a wide range of correlates representing multiple levels of influence as postulated by social ecological theory [25,75]. Ecological models therefore represent a promising theoretical framework that could be used to investigate the correlates of AT in the African context. Moreover, the included studies have almost exclusively examined the trip to/from school, underscoring a clear need for future research examining other trips (i.e. to/from friends’ and relatives’ residence, shops, markets, water points [to collect household water], places of worship, parks, and sport fields) which may represent important opportunities for PA [24]. Depending on considerations such as distance and parental safety concerns, walking or cycling to/from such destinations may also be more feasible than AT to/from school [76]. In addition, the included studies provided no information on travel patterns of children below 9 years of age, suggesting an important research gap in Africa. Information on travel behaviours among younger children (5–8 years) could help identify age-related variations in PA levels of youth in the African region.

In contrast with a previous systematic review [14], the African studies showed inconsistent relationships between AT and PA levels, and no association between AT and cardiovascular fitness. It is worth noting that the small sample size (n = 30) and the exceptionally high PA levels and VO_2_max (73.9 ml∙kg^−1^∙min^−1^ for boys and 61.5 ml∙kg^−1^∙min^−1^ for girls) of the adolescents who participated in the studies by Gibson et al. [39] and Ojiambo et al. [42] may partially explain the lack of association. Furthermore, in these two studies, all participants walked or ran to/from school covering long distances (7.5 ± 3.0 km/day), so distance traveled may not explain variations in PA and fitness in this sample. Nevertheless, three studies found positive associations between AT and PA [38,41,43], and another noted that children traveling longer distances had higher HDL cholesterol levels [44].

### Studies reporting psychometric properties of travel behaviours measures

Fourteen studies examined the test-retest reliability of school travel questions and substantial to almost perfect agreement was noted. However, the two studies that employed questions referring to a broader range of destinations found lower reliability. School travel may be a routinized behaviour such that over time, no conscious decision-making is required by parents and children [12,13]. For instance, parents interviewed by Faulkner and colleagues [12] described school travel as a “*routine*”, “*involving no real thought*”, or something that is “*habitual because obviously it’s what we do all the time*”. The school trip is also mandatory for children and youth, at least in high-income countries where the psychometric studies took place. In contrast, discretionary travel may be less dependent on habits. This hypothesis could be verified with measures of habit strength, like the one proposed by Verplanken and Orbell [77]. It is also worth noting that reliability coefficients for school travel measures tended to be greater when the time interval between assessments was shorter. Longer intervals could increase the likelihood of using different travel modes due to external circumstances (i.e., trip chains, weather, being late). The transferability of these findings from high-income countries to the African context is unknown and warrant further investigation.

Substantial convergent validity was noted between children- and parent-reported school travel modes in most studies, although there were some exceptions [52,59]. However, studies that examined the concurrent validity between different measures found conflicting results. For example, Philippaerts et al. [60] reported no association between reported time spent engaging in AT and accelerometry-measured PA in Belgian youth. Accelerometers are known to widely underestimate PA during cycling [78], a very common school travel mode in Belgium [67]. The wearable camera protocol used by Kelly et al. [56] should not have been affected by this limitation; however, wide limits of agreement were reported between self-reported trip duration and camera-estimated duration. This suggests that children may provide inaccurate reports of school trip duration. In addition, Rodriguez et al. [62], found only moderate concurrent validity between reported trips and trips identified by a combination of GPS and accelerometers. Collectively, these findings emphasize a need for further research on the concurrent validity of travel behaviour measures, especially in developing countries where reporting bias may differ. Again, such investigations should examine travel behaviour beyond the trip to/from school.

While Murtagh et al. [13] found good internal consistency for their measure of the number of steps accumulated during the school trip, their approach may erroneously consider other forms of PA as AT because steps accumulated from early morning to school arrival were assumed to reflect travel behaviour. Nevertheless, this measure was significantly predicted by children’s intention and habit strength relative to engaging in AT. Rosenberg and colleagues [62] also noted that walking to/from school, shops and parks was associated with several perceived built environment constructs in the hypothesized direction.

Just as in the present systematic review, in a previous review examining the relationship between AT and health-related outcomes, we noted that few studies of AT to/from school reported the reliability and validity of their measures of travel behaviour [14]. This can raise concerns about methodological quality, the trustworthiness of the conclusions drawn and the quality of evidence; thus in the context of measuring AT behavior, it is desirable for authors to assess and report the psychometric properties of their instruments. However, the specific types of reliability and validity that should be reported depend on the instruments used by the researchers. For instance, measures of test-retest reliability and convergent validity are particularly important when the researchers rely on reported travel patterns. Additionally, examining the concurrent validity of reported behaviour with an objective measure may help minimize concerns related to recall and social desirability biases. When using protocols requiring judgment by research staff (i.e. direct observation, coding of GPS trips), researchers should report intra- and inter-rater reliability.

### Limitations and strengths

The following limitations should be considered when interpreting the present findings. First, all included African studies were cross-sectional so it was not possible to determine changes over time in children and youth’s travel patterns and thus to confirm the PA transition. Second, only one of the African studies provided details on the psychometric properties of its travel behaviour measures, and children’s travel to/from non-school destinations has been largely understudied. Third, given the relative novelty of AT research in Africa, it is possible that potentially-relevant literature was not indexed appropriately in major databases such as MEDLINE and Embase. However, articles published in several African journals that are not indexed in these databases were also consulted. Fourth, the majority of studies relied on child and/or parent reports which may be vulnerable to social desirability bias like reports of PA in general [7]. Fifth, most of the psychometric studies focused only on school trips, so limited information is available about the reliability and validity of measures of trips to/from other destinations.

This study had several strengths. To our knowledge this is the first systematic review examining travel behaviour in African children and youth as well as the psychometric properties of measurement tools. Second, the rigorous systematic review methodology including two search strategies designed with input from a professional librarian is a major strength of this report. Third, the quality of included articles was assessed in a standardized and replicable way. Fourth, the consideration of non-English publications minimized the risk that relevant literature would be omitted from the review. Indeed, two non-English papers were included [37,55]. Finally, this systematic review substantially contributes to the literature by 1) emphasizing the need for further investigation of AT in African children and youth; and 2) providing clear directions for the refinement of travel behaviour measurement tools as well as for their adaptation in African countries.

## Conclusion

In conclusion, the present systematic review shows that AT research in Africa is in its infancy. The best available evidence indicates that AT is less common in urban areas and among children attending higher SES schools, and population level rates of AT to/from school tend to be lower than those reported in Northern European countries. Together, these findings are supportive of the PA transition model proposed by Katzmarzyk and Mason [8]. Decreasing rates of AT and PA may contribute to an increased risk of non-communicable diseases in countries where health systems are undermined by limited resources and where the prevalence of infectious diseases remain high. Clearly, there is a need for further research investigating this topic among African children and youth. To date, most of the international literature on AT has focused on the trip to/from school using self-report measures that shows substantial reliability and convergent validity. Objective measures of travel behaviours such as GPS, wearable camera and direct observation have been used much less often. Future work examining their reliability and validity is warranted. However, only one of the included psychometric studies have been done in Africa, thereby emphasizing the need for replication in the African context. Finally, further research is needed to develop valid and reliable survey instruments to assess trips to/from other destinations than school (i.e. friends’ and relatives’ residence, parks, sport fields, and shops).
